# Editorial: Host genome and metagenome interactions for improved ruminant health and performance

**DOI:** 10.3389/fmicb.2023.1160074

**Published:** 2023-03-21

**Authors:** Annabelle Meynadier, Christel Marie-Etancelin, Nikola Palevich

**Affiliations:** ^1^GenPhySE, Université de Toulouse, INRAE, ENVT, F-31326, Castanet Tolosan, France; ^2^AgResearch Ltd., Hamilton, New Zealand

**Keywords:** ruminant, genome, metagenome, health, performance

The gut microbiota of ruminants plays a central role in the nutrition of its host: it directly affects his health and his ability to produce both products of interest for the human diet and undesirable products such as methane. Ruminants have co-evolved with microorganisms capable of degrading plant fibers into absorbable chemical compounds that are nutritional precursors or bioactive compounds: these microbiota are likely to directly influence the animals' traits in the same way that the animal's genome can. Numerous studies have shown links between production or health traits and the gut microbiota. Studies on ruminal microbiota also point out a significant effect of the “host” factor, and few publications reported results concerning the impact of host genetics on the composition of ruminal microbiota. The objective of this paper collection is to gather the literature on a new approach to animal nutrition aimed at adapting the diet to the genetics of the animals in order to optimize the synergy between the genome and the ruminal metagenome in the expression of animal traits. To do this, we need to understand the relationship between the ruminal microbiota and the host and how they interact in the expression of phenotypes of holobiont.

One way to study the importance of the dialogue between the host and its microbiota is through microbial transplantation. Huang S. et al. investigated the consequence of ruminal content transplantation in prepartum dairy cows. They showed that fresh or sterilized rumen fluid affected host metabolism, in particular amino acids, bile acids and fatty acids metabolism.

Amin et al. highlighted the link between metabolism and ruminal microbiota on the one hand and host metabolism on the other hand, and milk production in dairy cows. Differences in metabolic pathways are noted between low and high milk producing cows. These differences are consistent between the rumen, serum and milk compartments, highlighting an integrated functioning at the holobiont level.

The difficulty in understanding the relationship between the rumen microbiota and its host is the strong impact of diet on the rumen microbial community and its metabolism. The paper by Zhang et al. showed in particular the effect of the protein level in the Yaks diet on the ruminal microbiota and metabolome.

The nature of the food may also be important. If we stay with proteins, their degradability can strongly impact the ruminal microbiota and its activity. This was observed by Wang et al. when they replaced soybean meal with fermented soybean meal in the ration of dairy cows.

More globally, feeding systems affect the ruminal microbiota and its activities, and thus modulate the expression of phenotypes of interest as demonstrated by Huang C. et al. on the growth of young Yaks. In this study, the authors also demonstrated the impact of feeding on the development of the rumen and its functioning, and thus on the construction of the holobiont and its production level.

Studies on the ruminal micrbiota and its host are increasingly leading us to consider these two elements not as two separate entities but as two parts of the same entity: the holobiont. It is the overall functioning of the holobiont that will determine the characteristics of an animal. However, this functioning depends on the environment in which the holobiont evolves, in particular the food it receives. The holobiont must therefore be understood in connection with the environment in which it evolves, and the future challenge for researchers will be to study the interactions between them in order to find the holobiont best adapted to a given environment.

## Author contributions

All authors listed have made a substantial, direct, and intellectual contribution to the work and approved it for publication.

